# The rs2071559 AA *VEGFR-2* Genotype Frequency Is Significantly Lower in Neovascular Age-Related Macular Degeneration Patients

**DOI:** 10.1100/2012/420190

**Published:** 2012-08-01

**Authors:** Stefano Lazzeri, Paola Orlandi, Michele Figus, Anna Fioravanti, Elisa Cascio, Teresa Di Desidero, Elisa Agosta, Bastianina Canu, Maria Sole Sartini, Romano Danesi, Marco Nardi, Guido Bocci

**Affiliations:** ^1^Ophthalmology Unit, University Hospital, Via Paradisa, 2-56124 Pisa, Italy; ^2^Division of Pharmacology and Chemotherapy, Department of Internal Medicine, University of Pisa, Via Roma, 55-56125 Pisa, Italy

## Abstract

In this prospective, case-control genetic study, 120 consecutive neovascular age-related macular degeneration (AMD) cases and 78 controls were enrolled. Two SNPs (rs2071559 and rs1870377) of *VEGF-A* receptor-2 (*VEGFR-2*) gene were analyzed with the technique of Real-Time PCR to investigate a genetic link between AMD and *VEGFR-2* gene polymorphisms in Italian patients. The frequency of the *VEGFR-2* genotype rs2071559 AA was significantly lower (18.33%) in patients with AMD than in the control subjects (34.62%; *P* = 0.0095, chi-square test; *P*_corr_ = 0.038; OR = 0.42, 95% CI 0.22 to 0.82). In conclusion, although with the limitations of a small sample size and the few SNPs studied, this study demonstrates a lower frequency of *VEGFR-2* rs2071559 AA genotype in an AMD patient population, suggesting future studies on the role *VEGFR-2* SNPs.

## 1. Introduction

Age-related macular degeneration (AMD) is a leading cause of irreversible blindness in individuals over the age of 50 in the Western world [1]. In persons aged 65 years and older, early stages of AMD occur in more than 15%, and end stages of AMD affect more than 3% [2].

Age-related macular degeneration is a complex disease and its origin is largely unknown. Currently, there are some firmly established risk factors [3, 4]. It is clear from the results of multiple genetic screens that the underlying genetic cause of AMD involves multiple genes, risk factors, and interactions [5].

Vascular endothelial growth factor-A (VEGF-A), coded for by the *VEGF-A *gene located on chromosome 6p21.3 [6, 7], is a key mediator of angiogenesis and vascular permeability through the high-affinity receptor tyrosine kinases VEGFR-1 (Flt-1) and *VEGFR-2* (Flk-1, KDR) [7]. *VEGFR-2* is responsible for the majority of the angiogenic and permeability-enhancing effects of VEGF-A [8]. Thus, VEGF-A and *VEGFR-2* are logical candidates for genetically influencing AMD susceptibility, based on their functional relevance to AMD pathophysiology. Besides, in a large study testing the association of 8 candidate genes with AMD, *VEGF-A* proved to have the strongest association with the disease in both a family-based study and a case-control study [9]. Polymorphisms of the *VEGF-A* gene have been investigated in AMD, but still the data seem to be controversial [10]. Recently, Boekhoorn and colleagues [11] did not find any association between AMD and the 3 common functional polymorphisms of the *VEGF-A* gene in a prospective population-based cohort study. No association between AMD and 7 single-nucleotide polymorphisms (SNPs) of the *VEGF-A* gene, located in its promoter and coding regions, was reported for an Anglo-Celtic population [12]. On the contrary, in a study of English population, 1 of 14 SNPs of the promoter and coding regions of the *VEGF-A* gene was associated with the wet form of AMD [13]. Also in a Taiwan Chinese population has been found an association between SNPs of the promoter of *VEGF-A* gene and AMD [14]. Against an intensive interest on *VEGF-A* gene polymorphisms and AMD, very few data are currently available on *VEGFR-2* polymorphisms and AMD.

The present study aimed to determine if a genetic association exists between some *VEGFR-2 *genotypes and neovascular AMD in an Italian population of patients.

## 2. Materials and Methods

### 2.1. Statement of Ethics and Subjects

All participants were enrolled from the clinical offices of the Santa Chiara University Hospital, Pisa, Italy from 2007 until 2009. We certify that all applicable institutional and governmental regulations concerning the ethical use of human volunteers/animals were followed during this research. The tenets of the Declaration of Helsinki were followed and the Santa Chiara University Hospital medical ethics committee approved the study (EudraCT number 2007-004487-53). All participants signed an informed consent form and gave permission to retrieve information from medical records. Patients with a diagnosis of neovascular AMD were included. Controls were identified as having no signs of AMD and were recruited from the same locations (Table 1).

### 2.2. Age-Related Macular Degeneration Definitions

The study was based on data from routine medical records. Cases had presented clinically; by far the most likely reason for such a presentation is that they had AMD causing visual symptoms.

For the diagnosis of AMD, fundus examination and photography, OCT imaging (Stratus OCT, Carl Zeiss Meditec, Dublin, CA), and angiography with fluorescein and indocyanine (Heidelberg Engineering, HRA + OCT Spectralis, Dossenheim, Germany) were obtained of the macular area of each eye after dilation of the pupils with tropicamide 1%. These transparencies and digitized images were graded with X12.5 magnification according to the International Classification and Grading System [15] by the same trained professional classifying AMD following the Rotterdam Study AMD classification [16–18]: we categorized the range of AMD fundus signs into five mutually exclusive stages 0 to 4 [19]. No AMD was defined as stage 0, no signs of AMD at all or only hard drusen (<63 *μ*m). Stage 1 was characterized by soft distinct drusen (≥63 *μ*m) or only pigmentary abnormalities. Stage 2 was soft, indistinct drusen (≥125 *μ*m) or reticular drusen only, or soft, distinct drusen (≥63 *μ*m) with pigmentary abnormalities. Stage 3 was considered as soft, indistinct drusen (≥125 *μ*m) or reticular drusen with pigmentary abnormalities. Stage 4 was similar to late AMD, subdivided into dry (geographic atrophy) and wet (neovascular) AMD.

The disease in each person was classified according to the highest stage of AMD in either eye. In this study we considered 2 groups: no AMD group and wet AMD. Lesions that were considered to be the result of generalized disease, such as diabetic retinopathy, chorioretinitis, high myopia, trauma, congenital diseases, or photocoagulation for reasons other than for wet AMD, were excluded from the AMD diagnosis.

### 2.3. *VEGFR-2* Genotyping

Blood samples (3 mL) were collected in EDTA tubes at the time of the first visit. DNA extraction was performed using QIAamp DNA Blood Mini Kit (Qiagen, USA). Real-time PCR single-nucleotide polymorphism (SNP) analysis of *VEGFR-2 *−604A/G (rs2071559) and 1719A/T (rs1870377) was performed using an ABI PRISM 7000 SDS (Applied Biosystems, USA) and validated TaqMan SNP genotyping assays (rs2071559, C_15869271_10; rs1870377, C_11895315_20; Applied Biosystems). PCR reaction was carried out according to the manufacturer's protocol.

### 2.4. Statistical Analysis

Statistical calculations were performed using the GraphPad Prism software package, version 5.0 (GraphPad Software Inc., San Diego, CA). Tests for Hardy-Weinberg equilibrium and linkage disequilibrium among the two *VEGFR-2* loci whose gametic phase is unknown were performed using PHASE and Arlequin version 3.1 software. Chi-square test was used to compare genotype frequencies between AMD patients and control subjects. The level of significance was set at *P* < 0.05. Corrected *P* values were calculated by multiplying *P* by the number of alleles/genotypes compared [20]. Odds ratio (ORs) with 95% confidence intervals (CIs) were determined for disease susceptibility of specific genotypes in the polymorphism of the *VEGFR-2*.

Statistical analysis to define population sample size and statistical power was performed with PAWE software (PAWE version 1.2, February 2003 written by Derek Gordon assisted by Michael Nothnagel, Rockefeller University, New York, NY) [21–24]. To assess the effect of potentially confounding variables other than patient genotype, a multivariate logistic regression analysis was also used (Stata Statistical Software v 10.0; USA).

## 3. Results

No significant differences were found between AMD patients and controls regarding variables such as age, gender, and hypertension. Table 2 shows the frequencies of *VEGFR-2* SNPs in the case and control population, respectively. Allelic distributions for *VEGFR-2* rs2071559 and rs1870377 were in Hardy-Weinberg equilibrium in both the case and control populations (Table 2). Moreover, *VEGFR-2* rs2071559 and rs1870377 SNPs were in linkage disequilibrium with each other in both the case (*χ* ^2^ = 0.89518, *P* = 0.34408) and control populations (*χ* ^2^ = 0.41618, *P* = 0.51885).

Allele rs2071559 A was less frequent in the AMD patients (44.58%) than in the control subjects (57.05%) with a *P* = 0.0153 (Table 3, chi-square test, *P*_corr_ = 0.0612) and a calculated OR = 0.61 (95% CI 0.40 to 0.91). The frequency of the *VEGFR-2* genotype rs2071559 AA was 18.33% in patients with AMD, while in controls it was 34.62% with a statistically significant difference equal to a value of *P* = 0.0095 (*P*_corr_ = 0.038; chi-square test, Table 3) and a calculated OR of 0.42 (95% CI 0.22 to 0.82). The frequency of allele rs1870377 A was not significantly different in the AMD patients (26.25%) than in the control subjects (19.87%) with a *P* = 0.14 (Table 3, chi-square test). The frequency of the *VEGFR-2* genotype rs1870377 AA was 8.33% in patients with AMD, while in controls it was 3.85% without any statistically significant difference (*P* = 0.21, chi-square, Table 3). Sample size and the allele frequencies of the rs2071559 polymorphism provided a statistical power of 64% for allelic test. The multivariate regression model showed that the lower frequency of the genotype rs2071559 AA in AMD patients remained when adjusting for age, gender, diabetes, high blood pressure, high blood cholesterol, heart disease, smoking, and anticoagulation therapy.

## 4. Discussion

The results of this study show evidence of a lower frequency of a particular *VEGFR-2* genotype and neovascular AMD in our cohort of 120 Italian patients. Indeed, the significant negative relationship (OR < 1) between the rs2071559 AA *VEGFR-2 *genotype and AMD could open novel perspectives in the etiology and in the estimate of risk factors in AMD. *VEGFR-2*, a tyrosine kinase receptor, is responsible for the main VEGF-A effects such as the proangiogenic and permeability processes [6]. At the moment, few studies of gene association have been performed for VEGF-A receptor genes; polymorphisms of *VEGFR-2 *were reported to be associated with sarcoidosis [25] and coronary heart disease [26]. Recently, a pioneering retrospective study on neovascular AMD cohort and *VEGFR-2* SNPs has been published [27]. The authors did not find any association for *VEGFR-2* SNPs by allele or genotype analysis, although a haplotype analysis did show a single rare haplotype to be mildly associated with AMD [27]. Which were the criteria for the selection of the *VEGFR-2* SNPs? Considering the potential impact on gene expression, SNPs located in promoter region of *VEGFR-2 *gene were considered. Moreover, the previous study by Fang et al. [27] did not investigate the promoter polymorphism rs2071559 (−604 A/G); thus in the present study, we chose this SNP among the polymorphisms previously associated with human diseases [25, 26, 28]. Moreover, we decided to include in our research also the *VEGFR-2* SNP rs1870377 because it has been significantly associated to other diseases. However, the rs1870377 was already studied in AMD patients without any significant results [27]. Our data confirmed the lacking of association between the rs1870377 and AMD, whereas, interestingly, we find out that the *VEGFR-2* rs2071559 AA genotype was significantly less frequent in AMD patients (*P* = 0.0095, *P*_corr_ = 0.038). However, no data are currently available on the VEGFR-2 protein concentration in endothelial cells of AMD patients carrying the genotype rs2071559 AA compared to normal controls carrying the AG/GG genotypes. It has been previously demonstrated that patients with coronary heart disease bearing the rs2071559 A allele exhibited higher transcription activity of *VEGFR-2* gene [26]. This higher transcription has indirectly been confirmed by the study of Hansen et al. [29] that showed a median protein concentration for the GG genotype (68 pg/mg) lower than the AA/AG genotypes (99 pg/mg) in 110 colorectal cancers. However, in contrast with what was speculatively expected, the same authors found out that the rs2071559 AA/AG genotypes were associated with a significantly lower microvessel density (*P* = 0.009) and with an improved survival of patients [29], despite a higher presence of *VEGFR-2* protein. Therefore, we cautiously hypothesize that the rs2071559 AA genotype could be associated to a lower risk to develop a pathologic microvessel formation even if in presence of higher levels of VEGF-A, causing less neovascular lesions typical of AMD. In light of these previous findings and our promising data, it is important that a large-scale prospective study will be conducted in order to improve our knowledge on this association. Indeed, a limitation of our study is the relatively small number of subjects recruited and the few number of investigated SNPs. Surely, future investigations should include more samples and also other *VEGFR-2* SNPs, such as the rs2305948 (1192C/T), in order to have a more complete picture of the role of *VEGFR-2* polymorphisms in AMD. The required number of subjects for 80% power is over 240 (affected) and 156 (unaffected), depending on the different allele frequencies of the SNP studied. However, our study maintains intact its exploratory importance because of the statistical restrictive approach to the data and for the novelty of its findings.

In conclusion, this study demonstrates the importance of *VEGFR-2 *SNPs in a neovascular AMD Italian cohort. We found pieces of evidences that polymorphisms in *VEGFR-2 *gene are significantly associated to neovascular AMD, at least in this Italian population, confirming that VEGF-A and its main receptor are implicated in the pathophysiology of AMD. As faithful predictors of response to antiangiogenic treatment in AMD have yet to be identified [30], the analysis of rs2071559 *VEGFR-2* gene polymorphisms could represent a promising options for those patients treated with ranibizumab or pegaptanib. Further studies aimed at confirming the influence of this polymorphism on AMD patients candidate to an antiangiogenic therapy are urgently needed.

## Figures and Tables

**Table 1:** Characteristics of patients.

Variable	Control	Wet AMD
Patients	78	120
Bilateral (*n*/%)	/	57/47.5%
Age		
(Yrs)	74.3 (DS 6.5)	80.2 (DS 8.9)
Range	65–90	53–99
Median	73.5	81.5
Gender		
F (*n*/%)	46/59%	61/50.8%
M (*n*/%)	32/41%	59/49.2%
Smoking		
Affected (*n*/%)	42/54%	70/58%
Diabetes mellitus		
Affected (*n*/%)	8/10.2%	11/9.2%
High blood pressure		
Affected (*n*/%)	31/39.7%	61/50.8%
High blood cholesterol		
Affected	5/4.7%	8/6.7%
Heart disease		
Affected	18/23.1%	29/24.2%
Anticoagulation therapy		
With therapy	23/29.5%	25/20.8%

**Table 2:** Frequency distributions of the analyzed *VEGFR-2* gene polymorphisms in 78 Controls and 120 AMD patients.

Controls					
Position	Genotype	*n* (%)	Allelic frequency *n* (%)	*χ* ^2^	Hardy-Weinberg equilibrium
	AA	27 (34.62)	89A (57.05) 67G (42.95)		
rs2071559	AG	35 (44.87)		*χ* ^2^ = 0.555	*P* = 0.456
	GG	16 (20.51)			

	AA	3 (3.85)	31A (19.87) 125T (80.13)		
rs1870377	AT	25 (32.05)		*χ* ^2^ = 0.003	*P* = 0.954
	TT	50 (64.10)			

AMD patients					

	AA	22 (18.33)	107A (44.58) 133G (55.42)		
rs2071559	AG	63 (52.50)		*χ* ^2^ = 0.568	*P* = 0.493
	GG	35 (29.17)			

	AA	10 (8.33)	63A (26.25) 177T (73.75)		
rs1870377	AT	43 (35.83)		*χ* ^2^ = 0.666	*P* = 0.414
	TT	67 (55.84)			

**Table 3:** Differences of *VEGFR-2* alleles and genotypes between controls and patients.

*VEGFR-2* SNPs	Controls	AMD	Total
rs2071559^∗^			
A	89 (57.05%)	107 (44.58%)	196
G	67 (42.95%)	133 (55.42%)	200

Total	156 (100%)	240 (100%)	396

rs1870377			
A	31 (19.87%)	63 (26.25%)	94
T	125 (80.13%)	177 (73.75%)	302

Total	156 (100%)	240 (100%)	396

rs2071559^°^			
AA	**27** ** ** **(34.62%)**	**22** ** ** **(18.33%)**	49
AG/GG	51 (65.38%)	98 (81.67%)	149

Total	78 (100%)	120 (100%)	198

rs2071559			
GG	16 (20.51%)	35 (29.17%)	51
AG/AA	62 (79.49%)	85 (70.83%)	147

Total	78 (100%)	120 (100%)	198

rs1870377			
AA	3 (3.85%)	10 (8.33%)	13
AT/TT	75 (96.15%)	110 (91.67%)	185

Total	78 (100%)	120 (100%)	198

rs1870377			
TT	50 (64.10%)	67 (55.84%)	117
AT/AA	28 (35.90%)	53 (44.16%)	81

Total	78 (100%)	120 (100%)	198

^∗^ *P* = 0.0153, *χ* ^2^, *P*_corr_ = 0.0612.

^°^ *P* = 0.0095, *χ* ^2^, °*P*_corr_ = 0.038.
